# Data on the microstructure and deformation of Fe_50_Mn_25_Cr_15_Co_10_N_x (x=0∼1.6)_ supporting the modifications of partial-dislocation-induced defects (PDIDs) and strength/ductility enhancement in metastable high entropy alloys

**DOI:** 10.1016/j.dib.2020.106713

**Published:** 2021-01-05

**Authors:** Byung Ju Lee, Jae Sook Song, Won Jin Moon, Sun Ig Hong

**Affiliations:** a Department of Advanced Materials Engineering, Chungnam National University, Daejeon 34134, South Korea; b Korea Basic Science Institute, Gwangju 61186, South Korea

**Keywords:** Partial-dislocation, Stacking fault, High-entropy alloy (HEA), Nitrogen, ε-martensite, Deformation twin, Strain rate sensitivity, Strain hardening

## Abstract

The data presented in this article are related to a research paper on the modification of deformed nanostructure and mechanical performance of metastable high entropy alloys (HEAs) [1]. Fe_50_Mn_25_Cr_15_Co_10_ alloys with and without nitrogen were synthesized in a vacuum induction furnace using pure metals of 99.99% purity and FeCrN_2_ as nitrogen source. The nitrogen content was determined by Leco O/N-836 determinator for nitrogen-doped alloys. Transmission electron microscopy (TEM) were carried at 200 kV equipped with energy dispersive spectroscopy (EDS). Tensile testing was performed at room temperature. The strain rate jump tests were conducted by changing the strain rate between 10^−3^ and 10^−2^ s^−1^ to measure the strain rate sensitivity. The nanostructural evolutions by deformation including extended stacking faults (ESFs), ε-martensite and twins were examined using EBSD and TEM for the annealed samples and those strained to different strain levels. The role of partial dislocations on the formation of various PDIDs were analysed and the energies stored as deformed nanostructure (ESDN) after the PDID band formation were used to predict the evolution of various nanostructure with strain. The data and approach would provide a useful insight into the nanostructural evolution in metastable high entropy alloys.

## Specifications Table

**Table:** 

Subject	Metals and Alloys
Specific subject area	Microstructure and mechanical properties of high entropy alloys.
Type of data	Table, Figure
How data were acquired	EBSD, TEM, mechanical testing, stored energy calculation
Data format	Raw data: TEM images, EBSD images, Stress-strain curves, Analyzed: Stored energy calculation data
Parameters for data collection	-TEM observations were carried out using a JEOL 200C microscope (JEOL, Tokyo, Japan) at 200 kV. -Tensile tests were performed at room temperature using a United mechanical testing machine at a strain rate of 10^−3^S^−1^ and 10^−4^S^−1^. - The grain structure and phase distribution on the surface were characterized using an electron backscatter diffraction (EBSD) system (Oxford Instruments, UK).
Description of data collection	Metallographic samples were cut, ground, and electropolished. Nanostructures of alloys before and after deformation were observed using TEM of jet polished and thinned TEM foils. The tensile data were acquired by tensile testing dog-bone-shaped plate specimens.
Data source location	Institution: Chungnam National University City/Town/Region: Daejon Country: Republic of Korea
Data accessibility	Data are hosted with the article. The raw data on the strain jump tests are in the Mendeley Data repository. link: https://data.mendeley.com/datasets/vbvtxn7cs4/1
Related research article	Byung Ju Lee, Jae Sook Song, Won Jin Moon, Sun Ig Hong, Modifications of Partial-Dislocation-Induced Defects and Strength/Ductility Enhancement in Metastable High Entropy Alloys through Nitrogen Doping, Mater. Sci. Eng. A (2021) https://doi.org/10.1016/j.msea.2020.140684

## Value of the Data

• The datasets on the microstructural evolution and stress-strain responses of Fe_50_Mn_25_Cr_15_Co_10_, Fe_50_Mn_25_Cr_15_Co_10_N_1.0_ and Fe_50_Mn_25_Cr_15_Co_10_ N_1.6_ can be used for developing a strategy for modifying the phase metastability and the mechanical performance of high entropy alloys.
• The dataset on the relationship between the deformation homogeneity caused by high strain rate sensitivity and the nanostructural evolution in nitrogen-free and nitrogen doped alloys can be useful for researchers on alloy design and nanostructural analyses of metastable HEAs.
• The data of the energy storage on the PDID nanostrucure based on the energy criteria and the modification of the PDIDs would provide a useful basis for designing high entropy alloys and high entropy steels with the strength/ductility enhancement.

## 1 Data Description

**Fig. 1:**
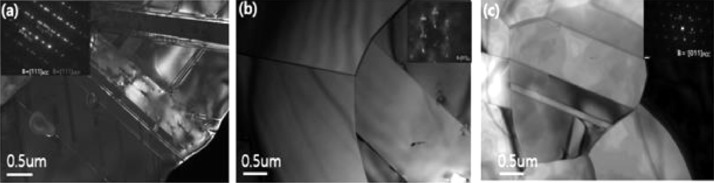
TEM micrographs of annealed Fe_50_Mn_25_Cr_15_Co_10_ (a), Fe_50_Mn_25_Cr_15_Co_10_N_1.0_ (b) and Fe_50_Mn_25_Cr_15_Co_10_N_1.6_ (c) HEAs.

Han et al. [2] reported that nitrogen addition in CoCrFeMnNi HEA enhanced the mechanical properties due to the increase of lattice frictional forces and the maintaining planar slip and twinning. Nitrogen doping is known to stabilize the face centered cubic (fcc) phase [3] and therefore modify the metastability of high entropy alloys [4,^5^,6]. Fig. 1 shows TEM micrographs of annealed Fe_50_Mn_25_Cr_15_Co_10_ (a), Fe_50_Mn_25_Cr_15_Co_10_N_1.0_ (b) and Fe_50_Mn_25_Cr_15_Co_10_N_1.6_ (c) high entropy alloys. Considerable fraction of thick thermally induced ε-martensite bands was formed in nitrogen-free Fe_50_Mn_25_Cr_15_Co_10_ alloys (Fig. 1(a)) and the fraction of annealing twin increased appreciably with increase of nitrogen content in Fe_50_Mn_25_Cr_15_Co_10_N_1.6_ (c). The absence of thermally induced ε-martensite bands in annealed nitrogen-doped alloys is consistent with the XRD data and EBSD data of the research article [reference 1]. The increase of annealing twins with increase of nitrogen content as shown in Fig. 1(b) and 1(c) is also supported by EBSD phase map (Fig. 1(d1) and Fig. 1(e1) of Reference 1). The large fraction of the thick thermally induced ε-martensite in Fig. 1(a) is attributed to the stability of hexagonal close packed (hcp) ε-martensite in nitrogen free Fe_50_Mn_25_Cr_15_Co_10_ alloy. The stability of the phases is dependent on the thermodynamic parameters [1,^7^,8].

**Fig. 2:**
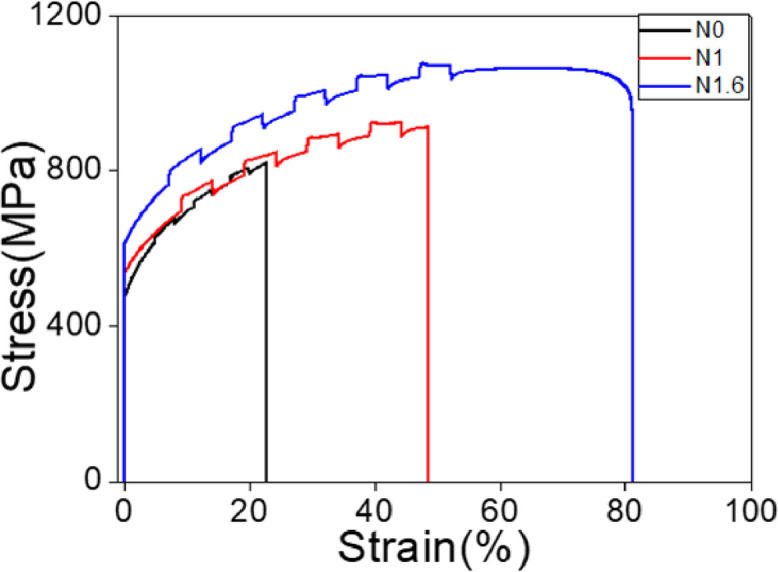
Stress-strain responses of Fe_50_Mn_25_Cr_15_Co_10,_ Fe_50_Mn_25_Cr_15_Co_10_N_1.0_ and Fe_50_Mn_25_Cr_15_Co_10_N_1.6_ HEAs with strain rate jumps between the strain rate of 10^−3^S^−1^ and 10^−2^S^−1^.

The strain rate jump tests were conducted by changing the strain rate between 10^−3^ s^−1^ and 10^−2^ s^−1^ to measure the strain rate sensitivity. Fig. 2 exhibits stress-strain curves with the strain rate periodically changed from 10^−3^ s^−1^ to 10^−2^ s^−1^ and then back to 10^−3^s^−1^ at room temperature. The strain rate sensitivity m was calculated based on the change of the flow stress obtained from the strain rate jump tests shown in Fig. S2 using the following equation [9,10]:

(1) $$m\;=\frac{\partial \;\text{ln}\;\sigma }{\partial \;\text{ln}\;\overset{\prime }{\varepsilon }}$$

where σ is the flow stress and $\overset{\prime }{\varepsilon }$ is the strain rate. Repetitive strain rate jumps between 10^−3^S^−1^ and 10^−2^S^−1^ were carried out. The strain rate sensitivity values calculated from the strain rate jump tests (Fig. 2) are summarized in Table 1. The strain hardening rates obtained from the stress-strain curves at the constant strain rates (Fig. 1 of Ref. 1) are summarized in Table 2. Interestingly, the strain rate sensitivity (SRS) increased significantly with increase of nitrogen content. The SRS at low strains of Fe_50_Mn_25_Cr_15_Co_10_N_1.6_ (∼0.024) was close to those of Cantor alloy [11], but it decreased more slowly than in Cantor alloy and the value (∼0.013) remained higher than Cantor at the strain of 0.3. The strain rate sensitivity of flow stress was found to decrease with strain as in equiatomic CoCrFeMnNi [10]. The enhanced ductility in Fe_50_Mn_25_Cr_15_Co_10_N_1.6_ can be attributed partly to the enhanced strain rate sensitivity.

**Table 1:** Variations of strain rate sensitivities with strain in Fe_50_Mn_25_Cr_15_Co_10_, Fe_50_Mn_25_Cr_15_Co_10_N_1.0_, Fe_50_Mn_25_Cr_15_Co_10_N_1.6_HEAs.

Strain	0.04	0.09	0.14	0.19	0.24	0.29	34
Alloys							
Fe_50_Mn_25_Cr_15_Co_10_	0.0111	0.0097	0.0089	0.0082			
Fe_50_Mn_25_Cr_15_Co_10_N_1.0_		0.0213	0.0179	0.0157	0.0122	0.0110	0.0107
Fe_50_Mn_25_Cr_15_Co_10_N_1.6_		0.0230	0.0190	0.0170	0.0140	0.0130	0.0126

**Table 2:** Variations of strain hardening rates with strain in Fe_50_Mn_25_Cr_15_Co_10_, Fe_50_Mn_25_Cr_15_Co_10_N_1.0_ and Fe_50_Mn_25_Cr_15_Co_10_N_1.6_ HEAs.

Strain	0.01	0.05	0.10	0.15	0.20	0.25	0.30
Alloys							
Fe_50_Mn_25_Cr_15_Co_10_	4031	2575	2251	2146	1900		
Fe_50_Mn_25_Cr_15_Co_10_N_1.0_	3984	2671	2306	2270	2244	2233	2185
Fe_50_Mn_25_Cr_15_Co_10_N_1.6_	4253	2923	2536	2467	2431	2408	2299

**Fig. 3:**
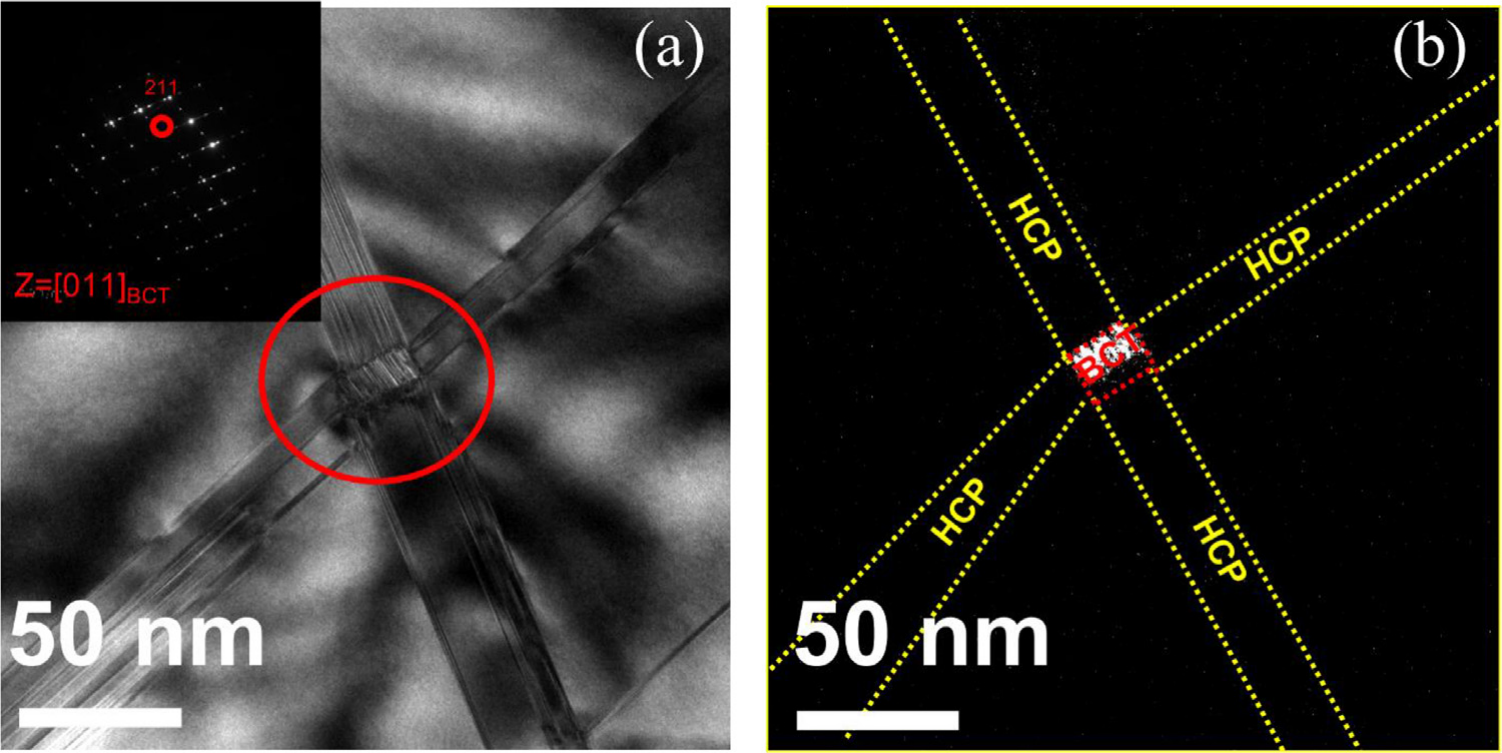
Bright field and dark field TEM images of nitrogen-free Fe_50_Mn_25_Cr_15_Co_10_ deformed to 20% strain. BCT α’-martensite was observed in the region of two crossing ε-martensite bands. The dark field image (b) exhibits the highlighted α’-martensite.

**Fig. 4:**
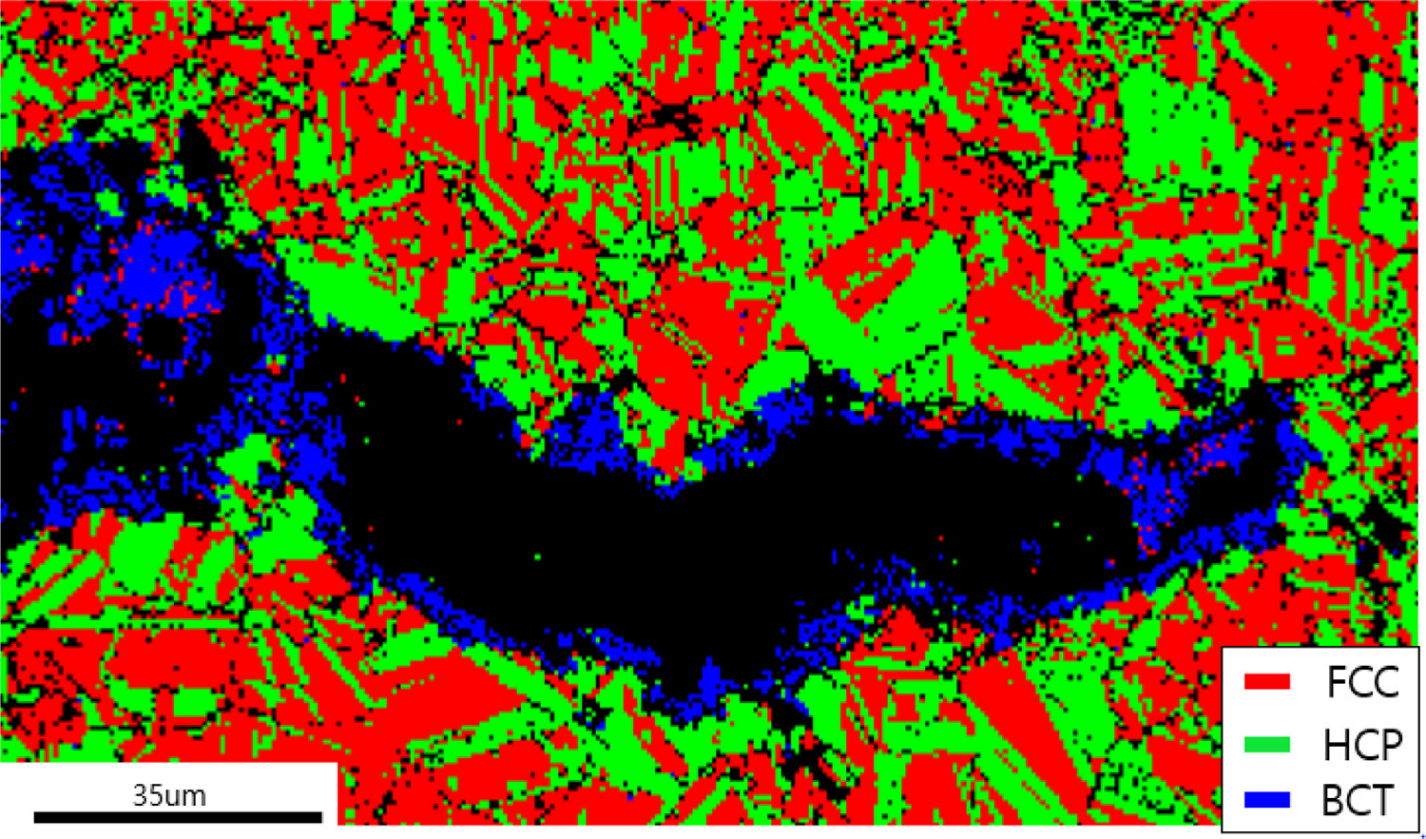
The presence of α-martensite along the propagating crack lowers the ductility in nitrogen free Fe_50_Mn_25_Cr_15_Co_10_.

Fig. 3 exhibits bright field and dark field TEM images of nitrogen-free Fe_50_Mn_25_Cr_15_Co_10_ deformed to 20%. The formation of formation α’-martensite (body centered tetragonal (bct)) phase was confimed in the region of intersecting ε-martensite bands at the strain of 0.2 in Fe_50_Mn_25_Cr_15_Co_10_. The dark field image (b) obtained from a spot from α’-martensite of the diffraction pattern (inset in (a)) proves that the highlighted phase in Fig. 3(b) is bct α’-martensite. The presence of larger α’-martensite was confirmed with the clear diffraction pattern from α’-martensite and reported in the related research paper on the modification of deformed nanostructure and mechanical performance of metastable HEAs [1]. The premature fracture of Fe_50_Mn_25_Cr_15_Co_10_ alloy at the strain of 0.25 suggests that the fracture crack may have nucleated at or near the region of α’-martensite because α’-martensites were usually formed in the localized strain region of intersecting ε-martensite. Fig 4 exhibits the presence of BCT α’-martensite near the propagating cracks and it may suggest that the presence of α’-martensite along the crack path were partly caused by the stress concentration [12] near the propagating cracks.

**Fig. 5:**
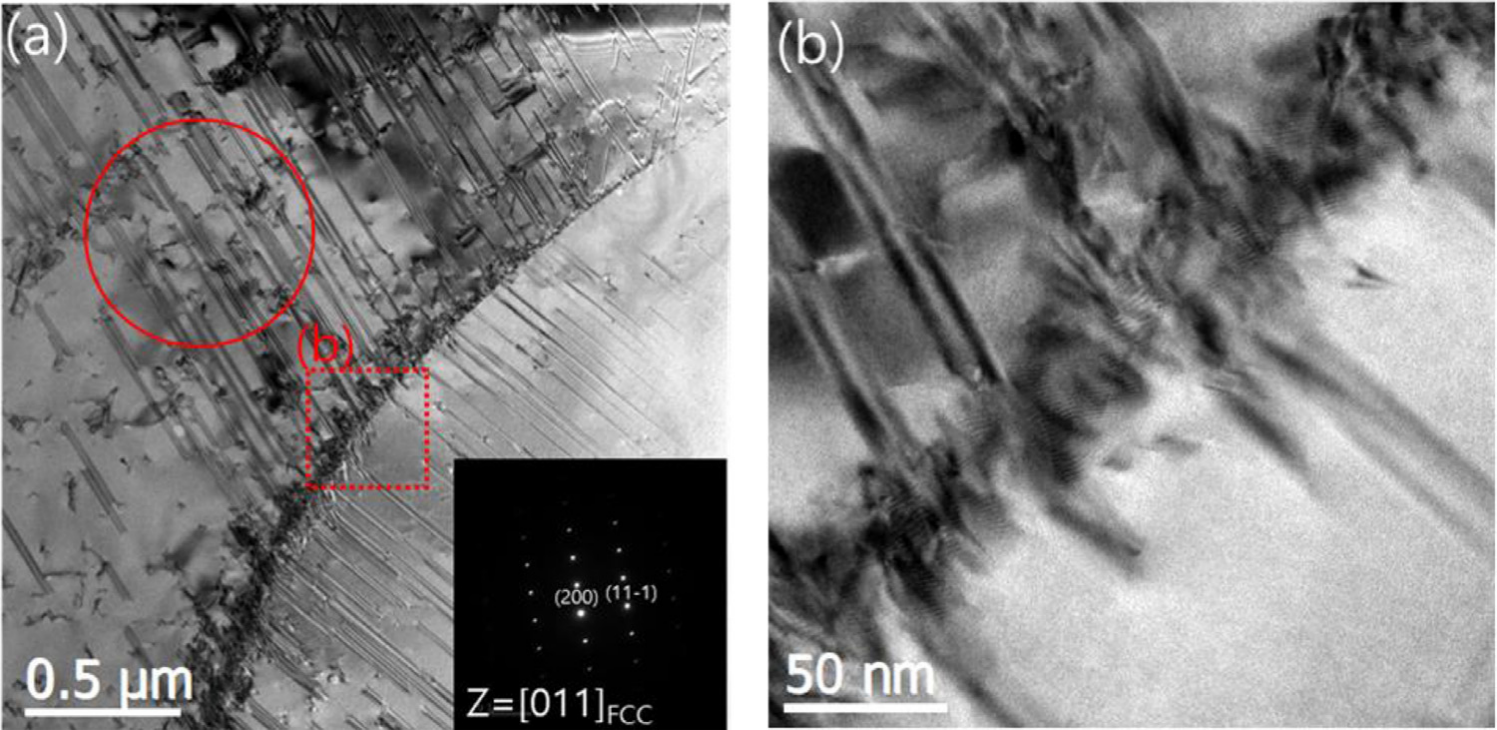
(a) Propagating ESFs impinged on the grain boundaries, (b) Enlarged view from the region enclosed by a red dotted square `b` shows the ESFs impinged on and nucleated from grain boundaries. (For interpretation of the references to color in this figure legend, the reader is referred to the web version of this article.)

Fig. 5 shows TEM images of Fe_50_Mn_25_Cr_15_Co_10_N_1.6_ deformed to 5%. At 5% strain, extended stacking fault (ESFs) were observed as shown in Fig S5(a). No diffraction spots from ε-martensite (hcp) were observed, suggesting they are ESFs [1]. The stacking faults in Fig. 5 were more widely extended than those observed in low stacking fault energy Cu-Al alloys [13,14] and other HEAs [6,15]. Propagating ESFs in Fig. 5 were observed to be impinged on the grain boundaries, and the enlarged view (Fig. S5(b)) from the region enclosed by a red dotted square in Fig. 5(a) shows the ESFs impinged on and nucleated from grain boundaries.

**Fig. 6:**
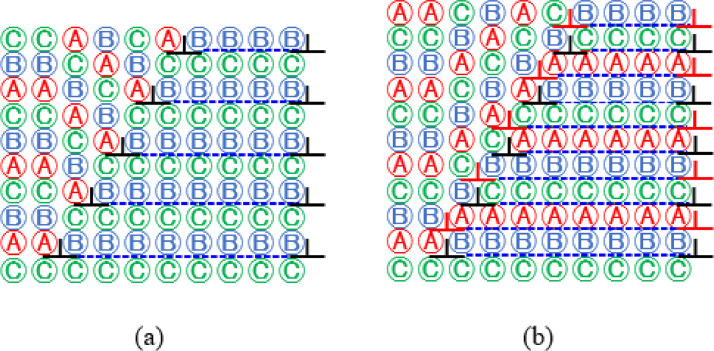
Transformation of ε-martensite (a) into deformation twin band (b) with the activation of new partial dislocations (red) on the unfaulted slip planes of ε-martensite band already formed by the activation of partial dislocations (black) every other plane. Deformation proceeds with the migration of partial dislocations in Fe_50_Mn_25_Cr_15_Co_10_N_x_ alloys. (For interpretation of the references to color in this figure legend, the reader is referred to the web version of this article.)

In Fig. 6, a schematic description for transformation of ε-martensite (a) into deformation twin band (b) with the activation of new partial dislocations (red) on the unfaulted slip planes of ε-martensite band already formed by the activation of partial dislocations (black) every other plane. Deformation proceeds with the migration of partial dislocations in Fe_50_Mn_25_Cr_15_Co_10_N_x_ alloys [See Fig. 7 of Ref. 1]. The stacking sequence correction by formation of parallel ESFs would facilitate and activate repetitive ESFs [1]. The reduction of stacking irregularities would be more effective for more closely spaced ESFs and this was suggested to promote the structure with closely spaced ESFs and increase the chance for the transition from closely spaced ESFs to deformation-induced ε-martensite and deformation twins with increase of strain [1].

The energies stored as deformed nanostructure (ESDN) for ESFs, ε-martensite or deformation twins in a grain with the size of 5 μm are summarized in Table 3 for comparison with those for grain size of 12 μm in Table 2 of Reference [1]. The differences of ESDN between ε-martensite and deformation twins in all three alloys for grain size of 5 μm were found to be greater than those of their counterparts with the grain size of 12 μm, suggesting the difficulty of deformation twinning with decrease of grain size. The ratios of grain boundary strain energy (GBSE) to partial dislocation induced defect energy (PDIDE) for deformation twinning of alloys with the grain size of 5 μm were also found to be far greater than their counterparts with the grain size of 12 μm (Compare the data in Table 3 to those of reference [1]).

**Table 3:** Energies stored as deformed nanostructure (ESDN) after the formation of PDID bands including bands of ESFs, ε-martensite or deformation twins in a grain with the size of 5 μm.

			Partial-dislocation-induced defect band energy (PDIDE) (J)						
Alloys(at%)	G.B strain energy(J) (GBSE)		Stacking Fault	Interfrace (FCC—HCP)	Twin boundary	△G (FCC→HCP)	E^str^	Total ESDN (J)	GBSE/ PDIDE
Fe_50_Mn_25_ Cr_15_Co_10_	SF	1.49 × 10^−17^	4.25 × 10^−17^					5.74 × 10^−17^	0.35
	Martensite	3.36 × 10^−17^		1.5 × 10^−16^		−1.12 × 10^−16^	6.44 × 10^−18^	7.86 × 10^−17^	0.75
	Twin	1.34 × 10^−16^			2.13 × 10^−17^			1.55 × 10^−16^	6.29

Fe_50_Mn_25_Cr_15_ Co_10_N_1.0_	SF	1.49 × 10^−17^	5.75 × 10^−17^					7.24 × 10^−16^	0.26
	Martensite	3.36 × 10^−17^		1.5 × 10^−16^		−9.69 × 10^−17^	6.44 × 10^−18^	9.31 × 10^−17^	0.56
	Twin	1.34 × 10^−16^			2.88 × 10^−17^			1.63 × 10^−16^	4.65

Fe_50_Mn_25_Cr_15_ Co_10_N_1.6_	SF	1.49 × 10^−17^	6.45 × 10^−17^					7.94 × 10^−17^	0.23
	Martensite	3.36 × 10^−17^		1.5 × 10^−16^		−8.99 × 10^−17^	6.44 × 10^−18^	1.00 × 10^−16^	0.51
	Twin	1.34 × 10^−16^			3.23 × 10^−17^			1.66 × 10^−16^	4.15

## 2 Experimental Design, Materials and Methods

Fe_50_Mn_25_Cr_15_Co_10_Nx (*x* = 0, 1.0, 1.6 at.%) alloys were cast in a vacuum induction furnace using pure metals of 99.99% purity and FeCrN_2_ as nitrogen source for nitrogen-doped alloys. Ingots were homogenized at 1323 K for 24 hrs in Ar atmosphere. Homogenized ingots were machined to cakes with the thickness of 15 mm (for nitrogen doped alloys) or 6 mm (for nitrogen-free alloys) and cold-rolled to sheets with 1 mm thickness and annealed at 1173 K for 1 h in an Ar atmosphere and air-cooled. Nitrogen-free alloys were found to be less ductile because of massive thermally and mechanically induced ε-martensite during rolling and the total cold rolling reduction was strictly controlled (47% cold rolling reduction compared to 92% reduction in nitrogen containing alloys) to avoid rolling cracks. The amount of FeCrN_2_ addition was adjusted to obtain the targeted nitrogen composition and the nitrogen content was determined to be 0.275 wt.% (1.0 at.%) and 0.412 wt.% (1.6 at.%) by Leco O/N-836 determinator, (Leco, Saint Joseph, MI, USA). Transmission electron microscopy (TEM) samples were prepared by mechanical grinding and electropolishing. TEM observations were carried out using a JEOL 200C microscope (JEOL, Tokyo, Japan) at 200 kV equipped with JED-2300T (JEOL, Tokyo, Japan) energy dispersive spectroscopy (EDS). Tensile testing was performed at an engineering strain rate of 10^−2^ s^−1^ ∼10^−3^ s^−1^ at room temperature. The strain rate jump tests were conducted by changing the strain rate between 10^−3^ and 10^−2^ s^−1^ to measure the strain rate sensitivity. The structural evolutions by deformation were investigated using EBSD and TEM for the strained and fractured tensile sample with different strain levels. The externally applied energy during quasi-static tensile testing was assumed to be converted to and stored as SFE, interface energy, twin boundary energy, grain boundary strain energy and/or phase transformation free energy of ε-martensite transformation (Eqs. (1), (2) and (3)). The energies stored as deformed nanostructure (ESDN) after the formation of PDID bands including bands of ESFs, ε-martensite or deformation twins was used to predict the nanostructural development based on the assumption that type of most dominant PDIDs were determined by the critical ESDN value required to form ESFs, ε-martensite or deformation twins.

## CRediT Author Statement

**Byung Ju Lee:** Data curation, Visualization, Writing - original draft. **Jae Sook Song:** Methodology, Validation, Investigation. **Won Jin Moon:** Methodology, Validation, Investigation. **Sun Ig Hong:** Supervision, Conceptualization. Writing - review & editing.

## Declaration of Competing Interest

The authors declare that they have no known competing financial interests or personal relationships which have or could be perceived to have influenced the work reported in this article.

## Appendix Supplementary materials

Image, application 1
